# Resting-state theta activity is linked to information content-specific coding levels during response inhibition

**DOI:** 10.1038/s41598-022-08510-8

**Published:** 2022-03-16

**Authors:** Charlotte Pscherer, Moritz Mückschel, Annet Bluschke, Christian Beste

**Affiliations:** Cognitive Neurophysiology, Department of Child and Adolescent Psychiatry, Faculty of Medicine, TU Dresden, Schubertstrasse 42, 01309 Dresden, Germany

**Keywords:** Cognitive control, Cognitive neuroscience

## Abstract

The neurophysiological processes underlying the inhibition of impulsive responses have been studied extensively. While also the role of theta oscillations during response inhibition is well examined, the relevance of resting-state theta activity for inhibitory control processes is largely unknown. We test the hypothesis that there are specific relationships between resting-state theta activity and sensory/motor coding levels during response inhibition using EEG methods. We show that resting theta activity is specifically linked to the stimulus-related fraction of neurophysiological activity in specific time windows during motor inhibition. In contrast, concomitantly coded processes related to decision-making or response selection as well as the behavioral inhibition performance were not associated with resting theta activity. Even at the peak of task-related theta power, where task-related theta activity and resting theta activity differed the most, there was still predominantly a significant correlation between both types of theta activity. This suggests that aspects similar to resting dynamics are evident in the proportion of inhibition-related neurophysiological activity that reflects an “alarm” signal, whose function is to process and indicate the need for cognitive control. Thus, specific aspects of task-related theta power may build upon resting theta activity when cognitive control is necessary.

## Introduction

The neurophysiological processes underlying response inhibition have been the subject of intense research^1–3^. Several studies have suggested that theta frequency activity plays an important role during inhibitory control^4–8^. This matches with concepts stating that successful processing of “surprise signals” is central to triggering response inhibition^9^ and that frontal midline theta activity serves to communicate such surprise signals^10^. Moreover, this is in line with overarching concepts suggesting that medial frontal theta activity orchestrates processes in different brain regions during cognitive control and integrates sensory and response-related processes^10^. Since theta oscillations orchestrate sensory and motor processes, both sensory and response-related processes are evident in neurophysiological correlates of inhibitory control, including theta power^11^.

While the role of theta band activity during response inhibition has been comparably well examined, the relevance of resting-state theta activity for neurophysiological processes during response inhibition has rarely been investigated. Yet, intra-cerebral recordings in humans and monkeys^12–14^ revealed that cortical structures involved in cognitive control-related theta band activity also produce endogenous theta band activity^15^. Together with the similarities between resting dynamics and activity during cognitive control^16–19^, this gives reason to assume that resting-state theta activity is important when trying to understand the neural dynamics of inhibitory control processes. Indeed, some findings suggest that resting theta activity at the frontal midline and in the middle frontal gyrus modulates response inhibition processes^20–22^. The existing evidence on the relationship between resting-state theta activity and the neurophysiological processes underlying response inhibition is insufficient to fully understand the role and relevance of resting-state theta-band activity for the neural processes underlying inhibitory control. Many questions remain to be addressed, such as whether a person’s resting-state theta activity varies with attentional or motivational states, or whether it is more of a trait marker. In this study, we focus on one very specific aspect among these issues: Given that different coding levels (i.e., sensory and motor processes) are crucial for response inhibition^23^, and since theta oscillations reflect these intermingled coding levels during response inhibition^11^, the question arises whether there are specific relationships between resting-state theta activity and sensory/motor coding levels during response inhibition.

Previous studies investigating the intermingling of sensory and motor coding levels during response inhibition^11,24^ did so by applying a temporal EEG signal decomposition method: residue iteration decomposition (RIDE)^25,26^. In response inhibition tasks (e.g., a Go/Nogo task), this method decomposes the EEG signal into two distinct clusters, the S-cluster and the C-cluster. The S-cluster reflects sensory processes, and the C-cluster reflects response selection and motor processes^11,25–27^. Using standard event-related potentials (ERPs), response inhibition processes are reflected by a characteristic Nogo-N2/Nogo-P3 complex^28^. Particularly the N2 time window has been suggested to reflect a mixture of sensory and response selection processes^29^, and evidence from signal decomposition research applying RIDE corroborated this notion^11,24,30^. Especially stimulus-related processes contribute to modulations of activity in the N2 time window during response inhibition^11^ and theta band activity is strongly pronounced in the N2 time window^15,31^.

When we use the term “theta activity” with respect to the present study, we refer to “theta power”. Based on the presented findings, we hypothesize that resting-state theta activity is correlated with the strength of neurophysiological processes reflecting stimulus-related codings, particularly in the N2 time window during inhibitory control. More specifically, we hypothesize that higher resting-state theta activity is related to stronger S-cluster amplitudes in the N2 time window during Nogo trials as well as to stronger task-related theta activity in the N2 time window. We expect these results especially at fronto-central electrodes. Response-selection-related processes in the N2 time window (reflected by the C-cluster) may show similar correlations, but modulations in the C-cluster are mostly observed in the P3 ERP time window^32^. Such a pattern of results would suggest that resting-state theta activity predicts the strength of the inhibition-related “surprise” signal and is specifically relevant for distinguishable subprocesses of the neurophysiological activity underlying response inhibition.

## Materials and methods

### Participants

We assessed *N* = 79 participants in the age range of 20–30 years (45 females; age: 24.96 ± 3.04; IQ: 110.95 ± 11.89). The sample partially overlaps with the samples of previous studies published by our work group on resting-state theta activity^21^. All participants were right-handed and did not report any neurological or psychiatric diseases. The absence of psychiatric disorders was determined by the *ASR/18-59* (Adult Self-Report;^33^). All subjects gave written informed consent prior to the start of the study procedures and received financial compensation upon completion of the testing. The local ethics committee of the TU Dresden approved the study.

### Assessment of resting-state theta activity

To measure the participants’ resting theta activity, we used the same setup as reported in previous studies^21,34^. We asked the participants to relax for two minutes with their eyes open while looking at a computer screen. During this two-minute time interval, the brain electrical activity was continuously recorded from an electrode placed at position Cz. An electrode on the right earlobe served as reference electrode and an electrode on the forehead was used as ground electrode. Eye movements were recorded with electrodes above and below the right eye. We applied an online 6th order Butterworth filter with a passband of 4–7 Hz to determine the theta frequency band activity. Since strong movement and blink artifacts can confound the estimation of theta activity, such artifacts were removed online. We determined specific bounds for the artifact correction. For the channels that recorded eye movements, the bound was set to 700 µV. This means that time segments with activity above 700 µV were removed automatically from the data for the online-estimation of theta frequency band activity. For the electrode Cz, the bound was defined at 100 µV. Furthermore, a minimum activity of 5 µV was required to prevent segment exclusion. For higher frequent artifacts in a frequency range of 25–35 Hz, the bound was set to 10 µV. In addition, we inspected the data manually to ascertain that the data was not affected by any other artifacts, which were not eliminated online. For statistical analysis, we used the mean theta power of this two-minute time interval as an estimate of resting-state theta activity.

### Task

**Figure 1:**
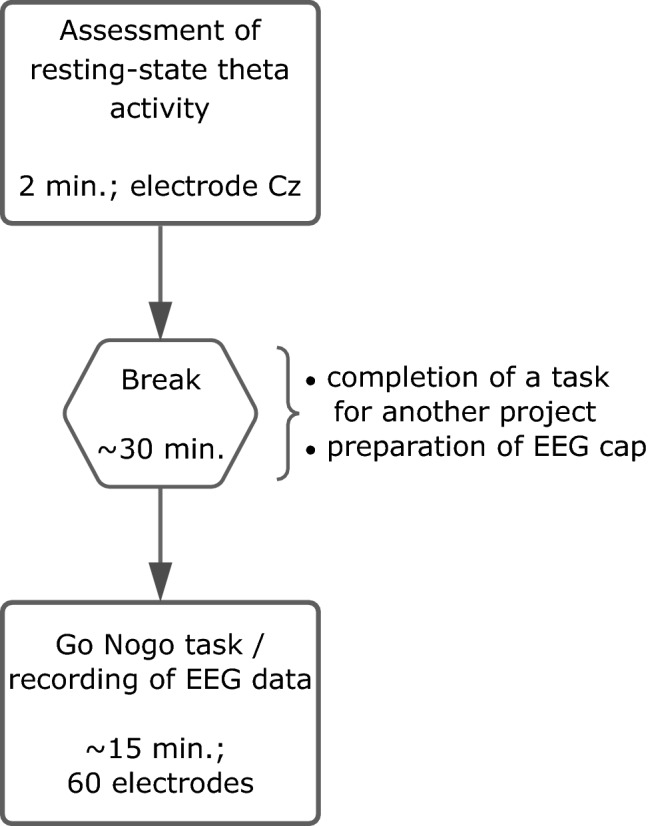
Flowchart showing the individual steps of the data collection and their duration.

After assessing resting theta activity, participants were asked to perform a standard Go/Nogo task^35^ to investigate their response inhibition performance. We used the software package “Presentation” (Neurobehavioral Systems) for this purpose. The experiment consisted of 168 Go trials and 72 Nogo trials. In case of a Go stimulus, the German word for “press” (i.e. “DRÜCK”) was presented; in case of a Nogo stimulus, the German word for “stop” (i.e. “STOPP”) was presented. Each stimulus was shown for 400 ms in white font on a black background of a 24-inch TFT screen. The inter-trial interval was jittered between 1700 and 2100 ms. The participants were instructed to react as quickly as possible by pressing a button with their right index finger each time a Go stimulus appeared. When a Nogo stimulus was shown, participants were required to inhibit their response. Go trials were classified as hits when a response occurred within 1200 ms after the stimulus onset. Nogo trials were coded as false alarms when a response occurred within 1500 ms after the stimulus onset. To increase the probability of premature responses in Nogo trials, 70% of the presented stimuli were Go trials and 30% were Nogo trials^36^. For the descriptive statistics, the response accuracy (given in percent) and the mean reaction time for hits (given in ms), as well as the mean false alarm rate (given in percent) were calculated for each participant. Figure 1 gives an overview of the individual steps of data acquisition and their duration.

### Event-related EEG recording and analysis

While the participants were performing the Go/Nogo task, an EEG was recorded from 60 equidistant Ag/AgCl electrodes mounted in an elastic cap (EasyCap Inc.) using the software BrainVision Recorder 2.1 (Brain Products Inc.). The reference electrode was located at θ = 90, ϕ = 90, the ground electrode at θ = 58, ϕ = 78. The electrode impedances were kept below 5 kΩ. During offline data processing with the software package BrainVision Analyzer 2.1 (Brain Products Inc.), the data was down-sampled from 500 to 256 Hz. We also applied an IIR bandpass filter from 0.5 to 20 Hz (slope: 48 dB/oct) and a notch filter of 50 Hz. Furthermore, we manually inspected the raw data and removed segments with muscular and technical artifacts. Subsequently, an independent component analysis (ICA, infomax algorithm) was carried out to identify and manually remove recurrent artifacts (e.g. eye movements, blinks, muscle, and pulse artifacts). Afterwards, the data were segmented and locked to the onset of the Go and Nogo stimuli. The length of each segment ranged from 2000 ms before stimulus onset to 2000 ms after stimulus onset. With these segments of a length of 4000 ms we obtained a sufficient time window for a reliable analysis of theta frequency oscillations using time–frequency analysis (see below). Only Go trials with a correct response given within 1200 ms after stimulus onset and Nogo trials without a response within 1500 ms after stimulus onset were included in the data analysis. Trials with an amplitude above 150 µV or below − 150 µV as well as trials with a maximum value difference of 150 µV in an interval of 200 ms or with activity below 0.5 µV in a 100 ms interval were removed from the data by an automated artifact rejection. A current source density (CSD) transformation was used to obtain a reference-free representation of the data^37^. The CSD transformation computes an estimate of the surface Laplacian of voltage values on the scalp. A spherical spline interpolation method is applied to estimate the entire voltage distribution. In addition to obtaining a reference-free distribution, volume conduction related smearing effects are also mitigated. Finally, we applied a baseline correction in the time window from − 200 ms to stimulus onset.

### Residue iteration decomposition (RIDE)

After preprocessing the EEG data, we decomposed the standard single-trial ERP signal into component clusters based on the latency variability applying the residue iteration decomposition (RIDE) method^25^. For this purpose, we used the RIDE toolbox, which is available online (http://cns.hkbu.edu.hk/RIDE.htm). Since a classical ERP signal consists of the average of various single trials with varying latencies, it is susceptible to smearing. By decomposing the signal based on the latency variability, RIDE prevents the signal from being smeared^25^. We decomposed each participant’s data into a component cluster with static latency (S-cluster) and into a component cluster with variable latency (C-cluster). These clusters are associated with different periods of cognitive processing^25^. Since the S-cluster is locked to the stimulus onset, it is associated with processes related to stimulus processing. The C-cluster is an intermediate cluster, which is neither locked to the stimulus nor to the response. Therefore, its latency is estimated initially and afterwards optimized by an iteration method^25,26^. The C-cluster is suggested to be related to cognitive processes regarding the stimulus–response transition and response selection^26^. RIDE provides the option to investigate a third component cluster, the R-cluster, which is locked to the response. Given that the focus of this study is on the Nogo trials, where participants were instructed not to respond, we did not examine the R-cluster. As pointed out by Ouyang et al.^38^ no reliable estimation of the R-cluster is possible in Nogo trials of a Go/Nogo since responses occur with too low frequency.

RIDE applies a time window function to obtain the wave shape of each component cluster. For this purpose, time intervals covering the expected occurrence of each component must be defined. For the S-cluster, we selected a time window from 200 before to 500 ms after the stimulus onset. For the C-cluster, the respective time interval was set from 150 to 900 ms after the stimulus onset. Using these time markers as initial estimates, RIDE splits the ERP signal into the individual components and adjusts the latencies iteratively. The software uses L1-norm minimization to obtain a median waveform for each component cluster for all trials^25^. Further methodological details regarding the RIDE method are explained by Ouyang et al.^25,26^. For the statistical analysis (correlation analysis), we used the average S-Cluster and average C-Cluster data for correctly rejected Nogo trials of each participant. For the descriptive neurophysiological data, we exported the peak N2- and P3-amplitudes at electrodes Cz and FCz of each participant using an automated peak detection function of the BrainVision Analyzer 2.1 (Brain Products Inc.). The electrodes were chosen since they showed the strongest activity in the scalp topographies. The negative peaks of the N2 component were defined in a time interval from 200 to 400 ms after the stimulus onset in the S-cluster. The positive peaks of the P3 component in the C-cluster were defined in the time window from 300 to 500 ms after stimulus onset.

### sLORETA

We used standardized low-resolution brain electromagnetic tomography (sLORETA) on the decomposed RIDE data for source localization^39^. sLORETA provides a single solution to the inverse problem without a localization bias^39–41^. The software divides the intracerebral volume into 6239 voxels with a 5 mm spatial resolution. It then calculates the standardized current density at each voxel. A realistic head model^42^ is used for this purpose, which is based on the MNI152 template^43^. We contrasted the voxel-based sLORETA images of the Nogo condition in both, the S-cluster and the C-cluster against zero. These comparisons were based on statistical non-parametric mapping (SnPM) and conducted with the sLORETA built-in voxel-wise randomization tests with 2000 permutations. Voxels with significant differences (*p* < 0.05, corrected for multiple comparisons) between the contrasted condition and zero were localized in the MNI-brain template^39^.

### Time–frequency decomposition

To analyze the task-related total theta power, we computed time–frequency (TF) analyses on the single trial RIDE data of each participant for both, the S-and the C-cluster. The total theta power is composed of phase-locked and non-phase-locked aspects of theta power. The analyses were conducted separately for Go and Nogo trials. We first applied Morlet wavelets (*w*):

$$w(t,f) = A\exp ( - t^{2} /2\sigma_{t}^{2} )\exp (2i\pi ft)$$

with the parameters *t* = time, *f* = frequency, $A = (\sigma_{t} \sqrt \pi )^{ - 1/2}$, $\sigma_{t}$ = wavelet duration, and *i* = $\sqrt { - 1}$. We used a Morlet parameter of f_0_/σ_*f*_ = 5, where f_0_ represents the central frequency and σ_*f*_ determines the Gaussian curve in the frequency domain. The time resolution (wavelet duration) can be calculated as 2σ_*t*_ and the frequency resolution (spectral bandwidth) can be calculated as 2σ_*f*_ for different f_0_. The two parameters σ_*t*_ and σ_*f*_ are related by the equation σ_*t*_ = 1/(2πσ_*f*_). The TF decomposition was conducted in 40 steps of 0.5 Hz in a frequency range from 0.5 to 20 Hz. For further analysis of theta band power, the time window was restricted to 200 ms before to 1000 ms after stimulus onset. The analysis was conducted for the electrodes FCz and Cz in the frequency band of 5 Hz. For the analysis of the descriptive neurophysiological data, the local maxima of the total theta power in the time window from 200 to 600 ms was determined, again at electrodes FCz and Cz at 5 Hz. This time window was chosen since maximal theta power related to response inhibition is usually detected within this time range^11,21,44^.

### Statistical analysis

We analyzed the descriptive behavioral and neurophysiological data with SPSS Statistics 26 and the correlation analyses of the neurophysiological data with MATLAB and the fieldtrip toolbox^45^. For the descriptive data of the resting theta activity and the task effects, the mean value and the standard deviation of the mean value are presented. Dependent samples t-tests were applied to test for differences in ERP peaks and peak total theta activity between the Go and Nogo trials. We further conducted correlation analyses by means of Pearson’s linear correlation coefficients to investigate the relationship between resting theta activity and task-related EEG activity. For this purpose, the subjects’ mean resting theta activity was correlated with each time point of the ERP data of correctly rejected Nogo trials data. This analysis was conducted for each of the 60 channels, separately for the S-Cluster and C-Cluster data. We computed correlations for each data point from 200 before stimulus onset until 1000 ms after stimulus onset, yielding 307 correlation coefficients per channel. We corrected the data channel by channel for multiple testing, applying the false discovery rate method^46^. Correlations with a *p*-value smaller than 0.05 after the correction procedure were considered significant. In the same way, correlation analyses between resting theta activity and the task-related total theta power were performed.

### Ethical statement

The study and all experimental procedures were approved by the ethics committee of the TU Dresden. The methods were carried out in accordance with the declaration of Helsinki. Written informed consent was obtained from all participants.

## Results

### Descriptive behavioral and neurophysiological data

The descriptive data showed that the participants had a mean resting theta activity of 3.42 ± 0.56 µV. During the Go/Nogo task, they showed a response accuracy of 99 ± 4% in Go trials and a mean hit reaction time of 359 ± 41 ms. The mean false alarm rate was 13 ± 11%. Resting theta activity did not correlate significantly with the hit rate in Go trials (*r*(77) = 0.04, *p* = 0.756), the hit reaction time (*r*(77) = 0.22, *p* = 0.150), or the false alarm rate in Nogo trials (*r*(77) = − 0.10, *p* = 0.756). All three correlations were calculated two-sided and the *p*-values were adjusted with a Bonferroni-Holm correction.

The peak EEG activity differed significantly between Go and Nogo trials. In the S-cluster, the N2 amplitude was significantly more negative during Nogo trials (Cz: − 27.25 ± 15.38 µV/m^2^; FCz: − 30.73 ± 15.34 µV/m^2^) than during Go trials (Cz: − 14.30 ± 9.48 µV/m^2^; FCz: − 14.06 ± 11.19 µV/m^2^), both at electrode Cz (*t*(78) = 10.52, *p* < 0.001) and at electrode FCz (*t*(78) = 12.72, *p* < 0.001). In the C-cluster, the P3 amplitude was significantly higher during Nogo trials (Cz: 32.63 ± 20.79 µV/m^2^; FCz: 31.48 ± 18.76 µV/m^2^) than during Go trials (Cz: 11.58 ± 14.09 µV/m^2^; FCz: 3.02 ± 17.77 µV/m^2^), both at electrodes Cz (*t*(78) = − 9.97, *p* < 0.001) and FCz (*t*(78) = − 14.26, *p* < 0.001). Furthermore, the event-related total theta power differed significantly between Go and Nogo trials. In the S-cluster, the peak total theta activity (selected in the time window from 200 to 600 ms after stimulus onset) was significantly higher during correctly rejected Nogo trials (Cz: 28,514.17 ± 22,609.67; FCz: 35,721.65 ± 23,394.13) than during Go trials (Cz: 17,682.22 ± 12,547.10; FCz: 23,872.31 ± 15,709.32) at electrodes Cz (*t*(78) = − 6.87, *p* < 0.001) and FCz (*t*(78) = − 7.59, *p* < 0.001). In the C-cluster (identical time window), the same pattern was observed: during correctly rejected Nogo trials, the peak total theta power was significantly higher (Cz: 25,918.79 ± 20,202.36; FCz: 31,581.49 ± 20,281.76) than during Go trials (Cz: 17,355.39 ± 12,583.40; FCz: 23,354.36 ± 14,901.77) at electrodes Cz (*t*(78) = − 6.32, *p* < 0.001) and FCz (*t*(78) = − 6.55, *p* < 0.001).

### Correlation analyses

For the correlation analyses, we correlated the participants’ mean resting theta activity with 307 time points of the event-related EEG signal and of the task-related theta power during correctly rejected Nogo trials, beginning from 200 before stimulus onset to 1000 ms after the stimulus onset. The correlations were computed for each of the 60 EEG channels, both for the S-cluster and the C-cluster.

**Figure 2:**
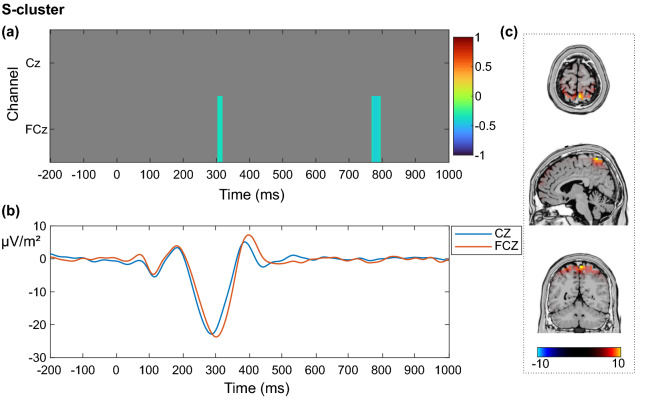
Correlation between resting theta activity and decomposed event-related potentials (ERPs) during correctly rejected Nogo trials in the S-cluster. (**a**) Correlation between resting theta activity and each time point of the EEG signal in the S-cluster from 200 ms before stimulus onset to 1000 ms after stimulus onset for the electrodes Cz and FCz. Only significant correlations with *p* ≤ .05 after a channel-by-channel correction with the false discovery rate method are shown. The correlation coefficient *r* is indicated by color. (**b**) Grand average of the participants’ ERPs in the S-cluster for the electrodes Cz (blue) and FCz (orange) from 200 ms before stimulus onset to 1000 ms after the stimulus onset. (**c**) sLORETA plots depicting activation differences in the superior parietal cortex (BA7) after contrasting the Nogo condition (correct rejections) in the S-cluster against zero. The color bar shows the critical *t*-values (*p* < .05, corrected for multiple comparisons).

In the S-cluster, the results revealed significantly negative correlations between resting theta activity and the event-related EEG signal at electrode FCz in the time intervals from 301 to 324 ms and from 770 to 793 ms after the stimulus onset (Fig. 2a).

**Figure 3:**
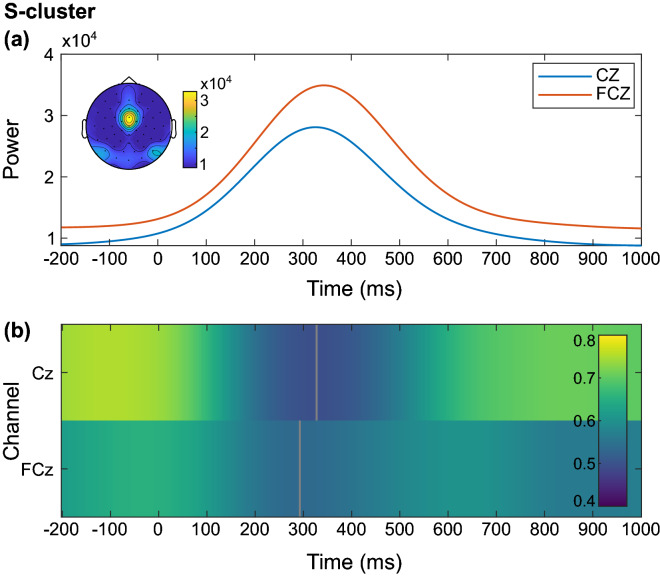
Time course of total theta power and correlation between resting and inhibition-related theta power in the S-cluster. (**a**) Participants’ grand average showing the course of inhibition-related total theta power in the frequency band of 5 Hz in the S-cluster from 200 ms before stimulus onset to 1000 ms after the stimulus onset at electrodes Cz (blue) and FCz (orange). The scalp topography corresponds to the time–frequency window of 344 ms and 5 Hz. (**b**) Correlation between resting-state theta activity and inhibition-related total theta activity in the S-cluster from 200 ms before stimulus onset to 1000 ms after stimulus onset for the electrodes Cz and FCz. Only significant correlations with *p* ≤ .05 after a channel-by-channel correction with the false discovery rate (FDR) method are shown. The grey areas were not significant after FDR correction. The correlation coefficient *r* is indicated by color.

Figure 2a depicts only the electrodes of main interest, i.e. the frontocentral channels Cz and FCz. For a full picture of the correlations with all 60 channels, please refer to the supplemental material (Fig. S1; https://figshare.com/articles/figure/Supplemental_material/19085111). A grand average of the S-cluster of all participants shows that the timing of the correlation in the earlier time window (301–324 ms) overlaps with the peak of the N2-component (Fig. 2b). The results imply that a higher resting theta activity is associated with more negative EEG activity at electrode FCz during the N2 time-interval in the S-cluster, i.e. with a stronger N2-peak. Since the second significant correlation is located in a very late time window (770–793 ms), we do not consider it as being related to the stimulus. Furthermore, such late time windows can hardly be differentiated from pre-trial activity related to the following stimulus^4^.The source localization analysis with sLORETA contrasted the Nogo condition in the S-cluster against zero and revealed activation differences in the superior parietal cortex (BA 7; Fig. 2c). After conducting the time–frequency analysis, visual inspection of the scalp topography showed that the task-related total theta power in the S-cluster during correctly rejected Nogo trials was highest at electrodes FCz and Cz (Fig. 3a).

Figure 3a shows the grand average of the course of total theta power at these electrodes with its peak in the N2 time window. The correlation analysis revealed significantly positive correlations between resting theta activity and the task-related total theta power in the entire time window (200 ms before stimulus onset to 1000 ms after stimulus onset) during correctly rejected Nogo trials in the S-cluster. Importantly, the correlation was weakest and even briefly not significant in the time interval overlapping with the peak of the N2 amplitude at electrode Cz (300–400 ms; Fig. 3b). The results show that in the S-cluster, participants’ total theta power is strongest during the N2 component but the overall strong correlation between resting and task-related theta activity is weakest in this time window.

**Figure 4:**
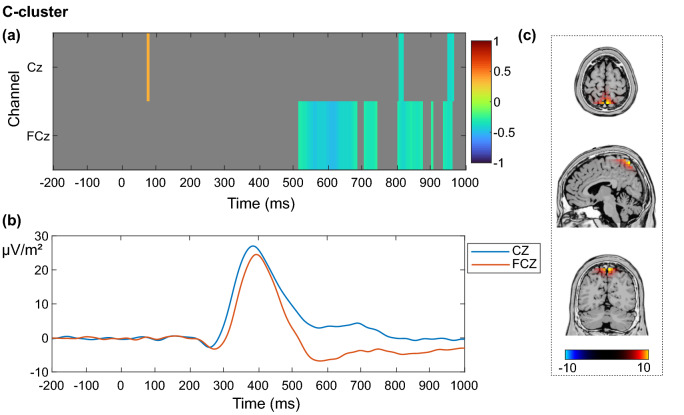
Correlation between resting theta activity and decomposed event-related potentials (ERPs) during correctly rejected Nogo trials in the C-cluster. (**a**) Correlation between resting theta activity and each time point of the EEG signal in the C-cluster from 200 ms before stimulus onset to 1000 ms after stimulus onset for the electrodes Cz and FCz. Only significant correlations with *p* ≤ .05 after a channel-by-channel correction with the false discovery rate method are shown. The correlation coefficient *r* is indicated by color. (**b**) Grand average of the participants’ ERPs in the C-cluster for the electrodes Cz (blue) and FCz (orange) from 200 ms before stimulus onset to 1000 ms after the stimulus onset. (**c**) sLORETA plots depicting activation differences in the superior parietal cortex (BA7) after contrasting the Nogo condition (correct rejections) in the C-cluster against zero. The color bar shows the critical *t*-values (*p* < .05, corrected for multiple comparisons).

In the C-cluster, the analysis yielded a significantly positive correlation between resting theta activity and the event-related EEG activity 70–80 ms after the stimulus onset at electrode Cz during correctly rejected Nogo trials. Furthermore, significant negative correlations were found in the time window from 523 to 680 ms after the stimulus onset at electrode FCz. Again, later correlations, from around 700 ms after stimulus onset on were not considered as being related to the stimulus. The correlation matrix of the C-cluster for the frontocentral electrodes Cz and FCz is presented in Fig. 4a.

**Figure 5:**
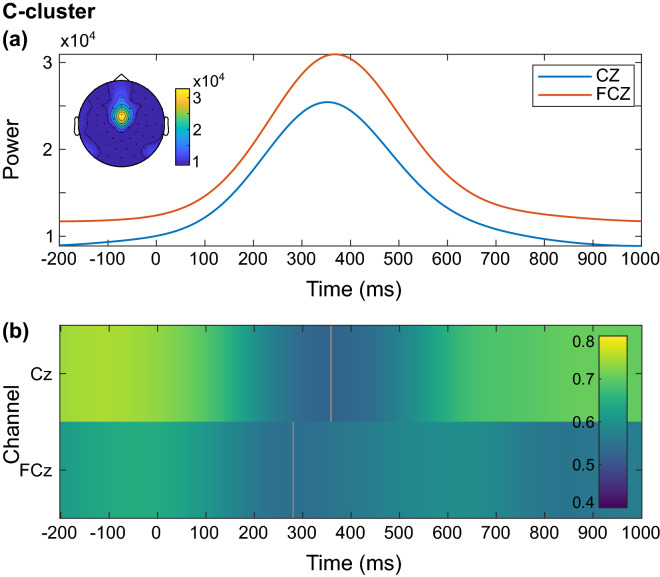
Time course of total theta power and correlation between resting and inhibition-related theta power in the C-cluster. (**a**) Participants’ grand average showing the course of inhibition-related total theta power in the frequency band of 5 Hz in the C-cluster from 200 ms before stimulus onset to 1000 ms after the stimulus onset at electrodes Cz (blue) and FCz (orange). The scalp topography corresponds to the time–frequency window of 367 ms and 5 Hz. (**b**) Correlation between resting-state theta activity and inhibition-related total theta activity in the C-cluster from 200 ms before stimulus onset to 1000 ms after stimulus onset for the electrodes Cz and FCz. Only significant correlations with *p* ≤ .05 after a channel-by-channel correction with the false discovery rate (FDR) method are shown. The grey areas were not significant after FDR correction. The correlation coefficient *r* is indicated by color.

As before, the full correlation matrix with all 60 channels is shown in the supplemental material (Fig. S2; https://figshare.com/articles/figure/Supplemental_material/19085111). It shows further negative correlations, similar to electrode FCz at electrodes Fz and F1, as well as late positive correlations at electrodes CP3, CP4 and P2. Figure 4b shows the average time series of all participants in the C-cluster. As can be seen, the negative correlations between resting theta activity and the event-related EEG signal in the frontocentral region coincide with the offset of the P3 component. The data imply that a higher resting theta activity goes along with a lower signal amplitude of the post-P3-offset at frontocentral electrodes in the C-cluster. The source localization analysis with sLORETA showed that the EEG signal during the Nogo condition in the C-cluster in comparison to zero was associated with activation differences in the superior parietal cortex (BA 7; Fig. 4c). As in the S-cluster, visual inspection of the scalp topographies after application of a time–frequency analysis revealed that the task-related total theta activity during correctly rejected Nogo trials in the C-cluster was strongest at electrodes FCz and Cz (Fig. 5a).

The course of the participants’ total theta power is depicted in Fig. 5a, peaking in a time window overlapping with the late N2- and early P3 components. The correlation analysis revealed significantly positive correlations between resting theta activity and the task-related total theta power in the entire time window (from 200 ms before stimulus onset to 1000 ms after stimulus onset) during Nogo trials in the C-cluster (Fig. 5b). At electrode Cz, the correlation was weakest (and very briefly non-significant) in the time window from about 300 to 400 ms, coinciding with the time window of the theta band power maximum as well as with the N2/P3 ERP complex. In summary, the results for the C-cluster indicate that the participants’ total theta power is strongest during the late N2/early P3 amplitude and that the overall strong correlation between resting and task-related theta activity is weakest in the time interval overlapping with this component.

As supplemental analyses, we computed correlation analyses between resting-state theta activity and the phase-locking factor (PLF) during correctly rejected Nogo trials in the S- and C-cluster. For details, please refer to the supplemental material (Figs. S3 and S4; https://figshare.com/articles/figure/Supplemental_material/19085111). Furthermore, we analyzed the correlation between resting-state theta activity and inhibition-related alpha activity to obtain a more detailed picture of the specificity of effects regarding the relationship between resting-state and inhibition-related activity. Please refer to supplemental Figs. S5–S8 for details.

## Discussion

The goal of the current study was to examine the interrelation of resting-state theta activity and event-related neurophysiological activity during response inhibition with particular focus on whether distinct fractions of information, coded simultaneously during response inhibition, are differentially linked to resting-state theta activity. The results show that this is the case.

In general, the results showed that the N2 and P3 amplitude, as well as the task-related total theta power were higher during Nogo trials than during Go trials, which replicates previous findings^21,28,36^. In the S-cluster, task-related total theta power peaked at the same time as the N2 amplitude peaked, which is in line with previous research suggesting that frontal midline theta underlies the N2 component^15,47,48^. Most importantly, after correlating the resting theta level with each time point of the EEG data from 200 ms before to 1000 ms after stimulus onset, it was evident that resting theta activity specifically correlated with the peak of the N2 amplitude in the S-cluster during successful response inhibition (i.e. correctly rejected Nogo trials). A higher resting theta level was associated with a stronger N2 peak. In contrast, in the C-cluster, the resting theta level was not related to the EEG data until the end of the P3 component. Only then, after the post-stimulus neurophysiological activity had abated and seemed to enter a resting or idling-like state, resting theta activity correlated with the EEG activity. Taking into account the literature suggesting that theta band activity may function as integration between the detection of the need for cognitive control and response implementation^10^, one might have assumed that resting-state theta activity should also be related to the C-cluster. Yet, our data showed that during the P3-peak in the C-cluster, no significant correlation between resting theta and task-related EEG data was evident. This seems logical though, since theta power is indeed generally evident during the N2 and P3 time window, but the N2 component is mainly driven by theta activity^15,31^, whereas slower delta power plays a major role in the (Nogo-) P3 time window^28,48–50^.

Thus, the data show that although processes related to stimulus processing and processes related to decision making or response selection take place simultaneously and are both coded in the N2 time window^11,29^, resting theta activity is very specifically associated with the fraction of neurophysiological activity that is related to stimulus processing. Why may resting theta activity be exclusively linked to stimulus-related processes during response inhibition?

EEG theta oscillations during cognitive control tasks may reflect *input integration* processes that lead to the detection of the need for cognitive control^15,34^. This may be possible because cognitive control-related EEG theta activity originates in cortical layers 2 and 3^14,15^, which yield a cellular architecture ideally suited to filter, amplify and integrate information^51,52^. This fits to other findings suggesting that theta frequency oscillations are suitable for information integration in large-scale networks^53^. The increase in task-related frontal midline theta activity is assumed to signal the realization of a need for cognitive control after a surprising event^10,15^ and thus acts as a stimulus-driven “alarm” or “surprise signal”^9,10^. Likewise, the N2 component usually occurs after a stimulus that is novel or requires cognitive control^29^. Our data support these theoretical and neurophysiological constructs, showing that resting theta activity is linked to the aspect of neurophysiological activity, which processes the stimulus and signals the need for inhibitory control. Yet, it is apparently not related to the entailed response selection, since no correlations with the C-cluster in the N2/P3 time window were obtained. Together with the finding that resting theta activity did also not correlate with behavioral performance, this suggests that participants with a higher resting theta level do detect the need for motor inhibition more strongly (i.e. using stimulus information). However, this “alarm” signal does not seem to be sufficient to enable better behavioral performance. Other processes seem to be important to react on the communicated “alarm” signal. Interestingly, the overall strong relationship (i.e. positive correlation) between resting and task-related theta activity during Nogo trials turned out to be weakest at the N2-peak and the task-related theta peak. It is remarkable, however, that the correlation disappeared only very briefly and was still predominantly significant during the N2 peak. This indicates that even during the possible coding of an “alarm signal” using stimulus-related information, aspects are coded in the theta frequency band that are similar to a resting-state. Thus, during response inhibition, theta activity reflects a dynamic that on the one hand acts as a “surprise signal”^9^, but on the other hand still reflects unspecific resting-state dynamics. The possible common ground between these at first sight different aspects in theta dynamics could relate to the relevance of the theta frequency band for input integration processes^15,34^. It may be speculated that resting-state theta activity could form the basis upon which control-related theta activity is built. In other words, it might be possible that resting-state theta activity provides the basis for a brain state that facilitates the detection of surprise signals allowing a more efficient initiation of inhibitory control processes. Therefore, resting theta power can serve as a predictor for the strength of the surprise signal. The results of the supplemental analyses (Analyses S3 and S4; https://figshare.com/articles/figure/Supplemental_material/19085111) support this suggestion revealing no significant correlation between resting-state theta and the PLF in both, S-and C-cluster. The PLF measures event-related phase consistency and therefore represents aspects of the evoked potential. Total theta power, which we examined in the main analysis, consists of phase-locked (evoked) and non-phase-locked (induced) aspects of theta power. Taking the results of the correlation analysis involving total theta power and the one involving PLF together supports the hypothesis, that resting-state theta is related especially to ongoing (induced) theta power during inhibition. It seems plausible that after an occurring stimulus, the evoked signal builds up on the ongoing (in our case resting-state theta) signal. Haegens and Golumbic review the state of research regarding the relationship between ongoing end evoked oscillations^54^. Our study is the first to show the link between resting theta activity and information content-specific processes (i.e. stimulus-related coding processes) during subsequent response inhibition.

Interestingly, a very similar result pattern has been shown in a previous study of our work group examining the role of resting theta activity during conflict monitoring, i.e. another domain of cognitive control^34^. The participants’ resting-state theta level was exclusively correlated to the stimulus-related coding level of the EEG signal, but not to the response-related coding level nor to behavioral performance^34^. Together, these findings suggest that resting-state theta activity plays a general role for the detection and processing of the need for cognitive control and seems not to be limited to one subdomain.

Considering the functional neuroanatomical level, the data show that the superior parietal cortex was activated during the S-cluster N2-peak in Nogo trials, i.e. at the time point when resting theta activity showed its weakest, but still significant correlation with task-related EEG activity in the S-cluster. Theta activity in this region^21^, as well as theta connectivity in the fronto-parietal network^55–57^ has been associated with various domains that are important for cognitive control: goal-directed attention, conflict processing, inhibition, as well as working memory capacity-related differences in cognitive control. Furthermore, several studies reported that the parietal cortex per se is involved in the perceptual processing of stimulus saliency^58–61^. Primate research suggests that the parietal cortex is involved in perception–action-integration^62^. Consistently, previous data have also suggested that superior parietal regions are involved in inhibitory control during the perceptual processing of inhibition-relevant stimuli^63^, especially when sensory information is complex or conflicting^64–67^. Together, these findings are in line with the current data showing that the superior parietal cortex is activated at the time point when the “alarm” signal processing the stimulus emerges. Although this region is active in both the S- and the C-cluster, only those processes in this region that are involved in stimulus processing (i.e. S-cluster processes) are linked to resting theta activity. The superior parietal cortex thus might be especially related to the input integration mechanisms underlying the “alarm” signal that communicates the need for cognitive control.

The current study sheds light on one particular aspect within a complex neurophysiological system. Many other questions still need to be addressed to obtain a broader framework. Since our study followed a hypothesis-driven approach, data analysis was limited to the theta band. A first exploratory outlook on more complex inter-relations between frequency bands is provided in the supplemental material (https://figshare.com/articles/figure/Supplemental_material/19085111). Analyses S5–S8 present correlations between resting-state theta, inhibition-related theta, and inhibition-related alpha activity. The results reveal significantly positive correlations between resting theta activity and inhibition-related alpha activity, which were smaller and less specific than with inhibition-related theta activity. Yet, these results give a first hint on the complexity of interrelationships between various frequency bands underlying inhibitory control processes. Future (exploratory) research should additionally include resting-state activity of other frequency bands to investigate whether variation in theta power during cognitive control is exclusively related to variations in theta activity at rest or rather to a more global change of several EEG bands at rest. Such analyses would provide further insights into how similar or distinct/independent resting and task-related theta power are. Additionally, future studies should consider investigating the interplay between various frequency bands at rest and their importance for cognitive control to gain further insight into which neurophysiological processes influence successful cognitive control at the performance level. To obtain a more comprehensive picture, the research question should be followed up with more complex tasks involving more demanding discrimination and more response options. It is also important that the role of resting-state theta activity is investigated from different angles. Motivational and attentional aspects should be considered to address the question of whether resting-state theta activity is a state marker or a trait marker, and to provide more detailed insights into how and whether resting-state theta forms a basis for cognitive control-related theta activity.

In conclusion, our data showed that resting theta activity is specifically correlated with the stimulus-related coding level of neurophysiological activity in the N2 time window during motor inhibition. In contrast, time ranges related to decision-making or response selection processes as well as behavioral inhibition performance were not associated with resting-state theta activity. Even at the peak of task-related theta power where task-related theta activity was the most dissimilar to resting theta activity, there was still a predominantly significant correlation between both types of theta activity. This indicates that aspects that are similar to resting dynamics are coded within the portion of inhibition-related neurophysiological activity, which functions as “alarm” signal processing the stimulus and recognizing the need for cognitive control. Thus, task-related theta power might build upon resting theta activity when cognitive control is necessary. Motivational and attentional aspects, as well as the interrelation with other frequency bands should be addressed in future research.

## Supplementary Information

Supplementary Information.

## Data Availability

The datasets generated and analyzed during the current study are available from the corresponding author on reasonable request.
