# Supplementary material for: Resting-state theta activity is linked to information content-specific coding levels during response inhibition
Source: Sci Rep. 2022 Mar 16;12:4530. doi: 10.1038/s41598-022-08510-8 (PMC8927579; doi:10.1038/s41598-022-08510-8)
Supplement: Supplementary file 1 — Supplementary Information. [file 41598_2022_8510_MOESM1_ESM.pdf]

Supplemental material

Resting-state theta activity is linked to information content-specific coding levels during response inhibition

Charlotte Pscherer, Moritz Mückschel, Annet Bluschke, Christian Beste

Supplemental Figure 1

S-cluster

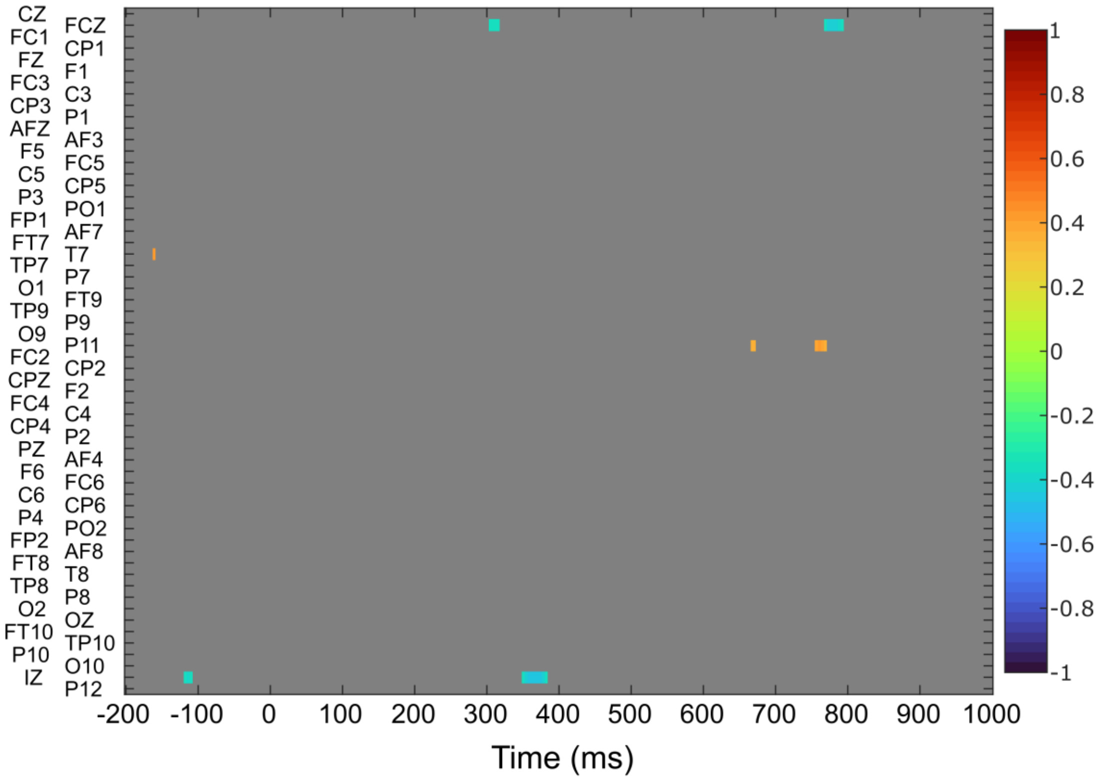

Figure S1

Correlation between resting theta activity and each time point of the EEG signal in the S-cluster from 200 ms before stimulus onset to 1000 ms after stimulus onset for all 60 channels. Only significant correlations with $p \leq .05$ after a channel-by-channel correction with the false discovery rate method are shown. The correlation coefficient r is indicated by color.

Supplemental Figure 2

C-cluster

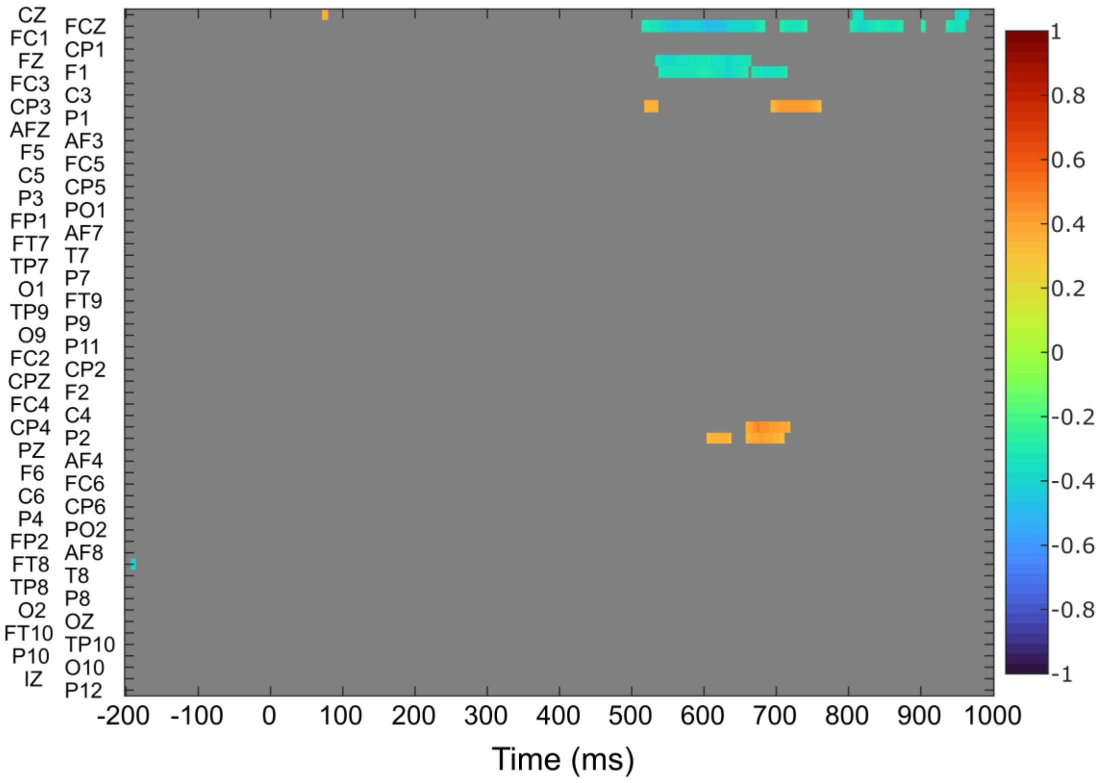

Figure S2

Correlation between resting theta activity and each time point of the EEG signal in the C-cluster from 200 ms before stimulus onset to 1000 ms after stimulus onset for all 60 channels. Only significant correlations with $p \leq .05$ after a channel-by-channel correction with the false discovery rate method are shown. The correlation coefficient r is indicated by color.

Supplemental Figure / Analysis 3

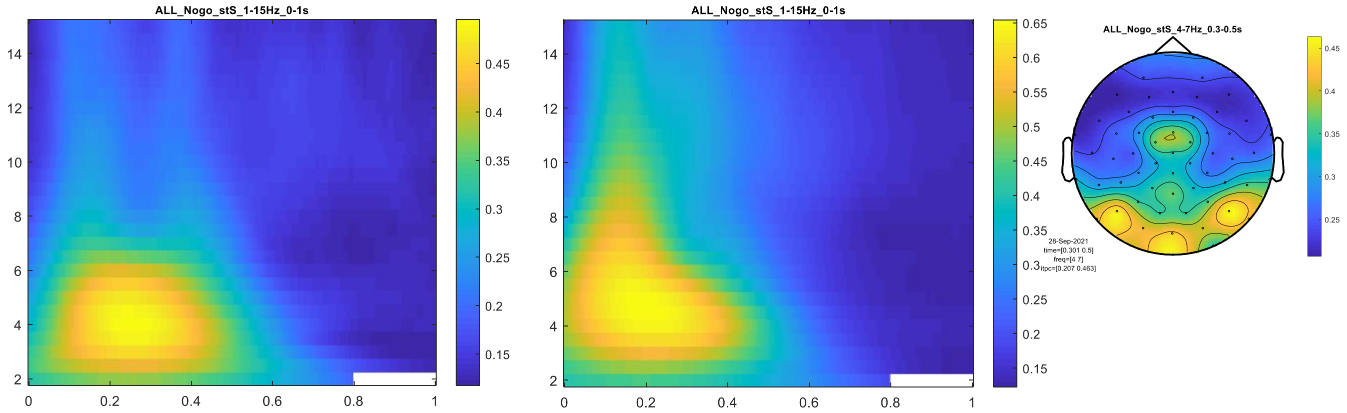

Figure S3
Phase-locking factor (PLF) during correctly rejected Nogo trials in the S-cluster. The value of the PLF is indicated by color. The left panel shows the PLF for channels Cz and FCz; the middle panel shows the PLF for channels P8, P7, O1, O2, and Oz. Channels were selected according to the topographic plot on the right.

Figure S3 shows the PLF during correctly rejected Nogo trials in the S-cluster. The PLF reached its maximum between 200 and 300 ms after stimulus onset. We computed a correlation analyses between resting-state theta activity and each time point of the phase-locking factor (PLF) in the S-cluster in the frequency band of 5 Hz from stimulus onset (0) to 1000 ms after stimulus onset for the channels Cz and FCz. A maximal correlation coefficient of -.25 was revealed. However, after a channel-by-channel correction with the false discovery rate method no significant correlations ($p \leq .05$) were evident.

Supplemental Figure / Analysis 4

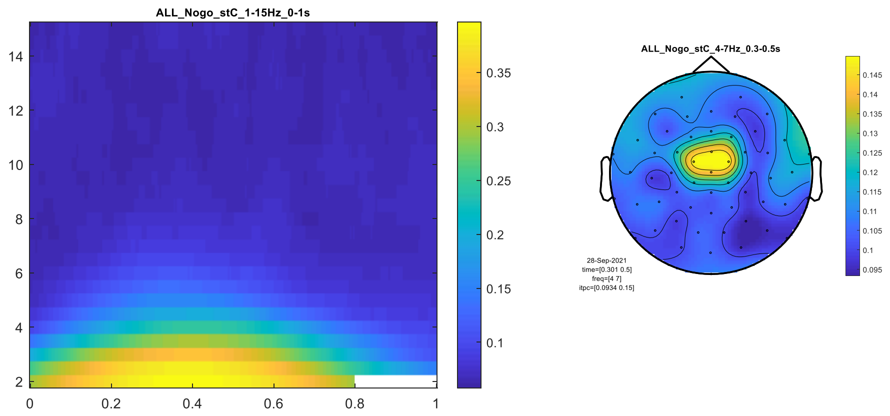

Figure S4

Phase-locking factor (PLF) during correctly rejected Nogo trials in the C-cluster. The value of the PLF is indicated by color. The left panel shows the PLF for channels Cz and FCz. Channels were selected according to the topographic plot in the right panel.

Figure S4 shows the PLF during correctly rejected Nogo trials in the C-cluster. The PLF reached its maximum between 300 and 400 ms after stimulus onset. We computed a correlation analyses between resting-state theta activity and each time point of the phase-locking factor (PLF) in the C-cluster in the frequency band of 5 Hz from stimulus onset (0) to 1000 ms after stimulus onset for channels Cz and FCz. A maximal correlation coefficient of .25 was revealed. However, after a channel-by-channel correction with the false discovery rate method no significant correlations ($p \leq .05$) were evident.

Supplemental Figure / Analysis 5

S-cluster

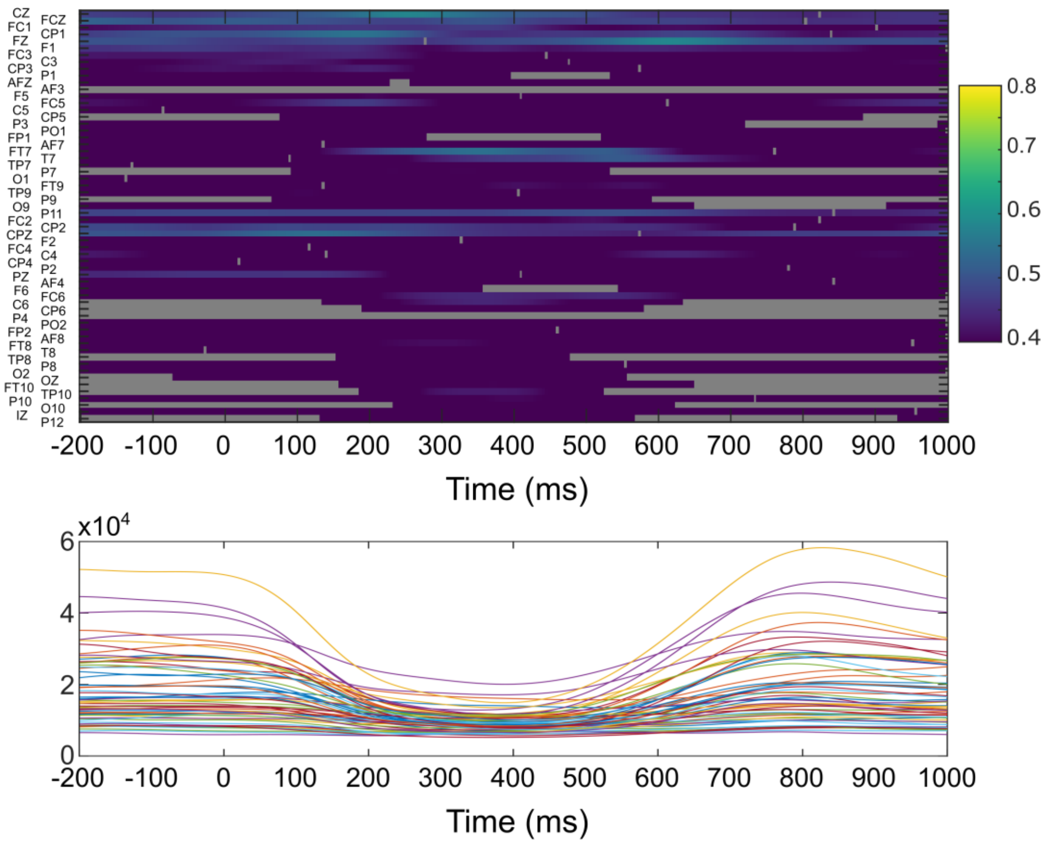

Figure S5

Correlation between resting theta activity and inhibition-related alpha power in the S-cluster. The upper panel shows the correlation from 200 ms before stimulus onset to 1000 ms after stimulus onset for all 60 channels. Only significant correlations with $p \leq .05$ after a channel-by-channel correction with the false discovery rate method are shown. The correlation coefficient r is indicated by color. The grey areas were not significant after FDR correction. The lower panel depicts the participants' grand average of the course of inhibition-related alpha activity (9-12 Hz) in the S-cluster from 200 ms before stimulus onset to 1000 ms after stimulus onset for all 60 channels.

Supplemental Figure / Analysis 6

C-cluster

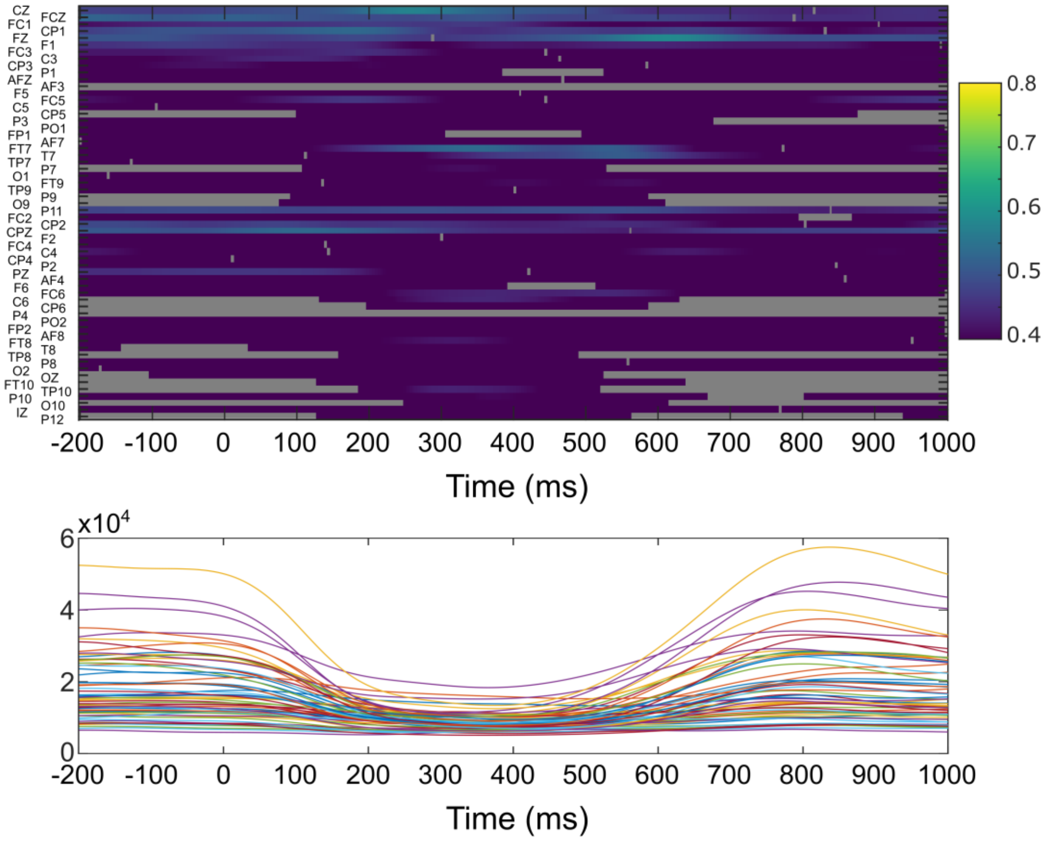

Figure S6

Correlation between resting theta activity and inhibition-related alpha power in the C-cluster. The upper panel shows the correlation from 200 ms before stimulus onset to 1000 ms after stimulus onset for all 60 channels. Only significant correlations with $p \leq .05$ after a channel-by-channel correction with the false discovery rate method are shown. The correlation coefficient *r* is indicated by color. The grey areas were not significant after FDR correction. The lower panel depicts the participants' grand average of the course of inhibition-related alpha activity (9-12 Hz) in the C-cluster from 200 ms before stimulus onset to 1000 ms after stimulus onset for all 60 channels.

Supplemental Figure / Analysis 7

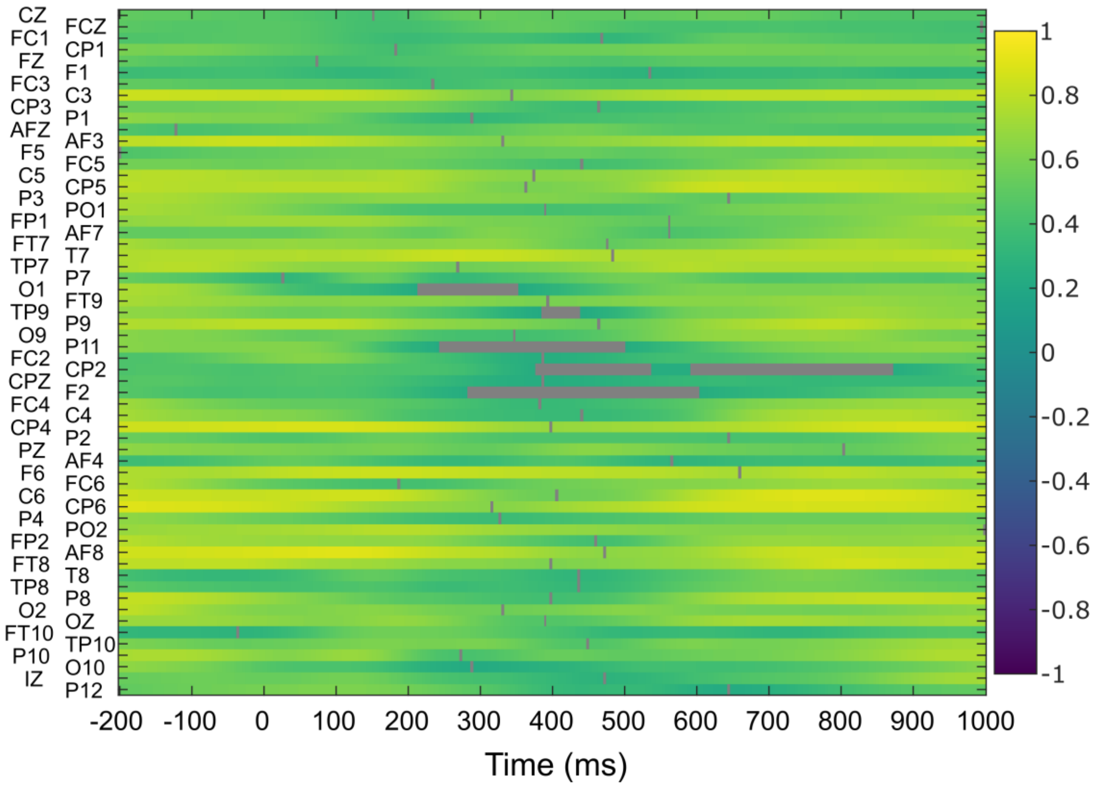

Figure S7

Correlation between inhibition-related theta and alpha power in the S-cluster. The figure depicts the correlation matrix for theta and alpha activity at each time point of the EEG signal in the S-cluster from 200 ms before stimulus onset to 1000 ms after stimulus onset for all 60 channels. Only significant correlations with $p \leq .05$ after a channel-by-channel correction with the false discovery rate method are shown. The correlation coefficient *r* is indicated by color. The grey areas were not significant after FDR correction.

Supplemental Figure / Analysis 8

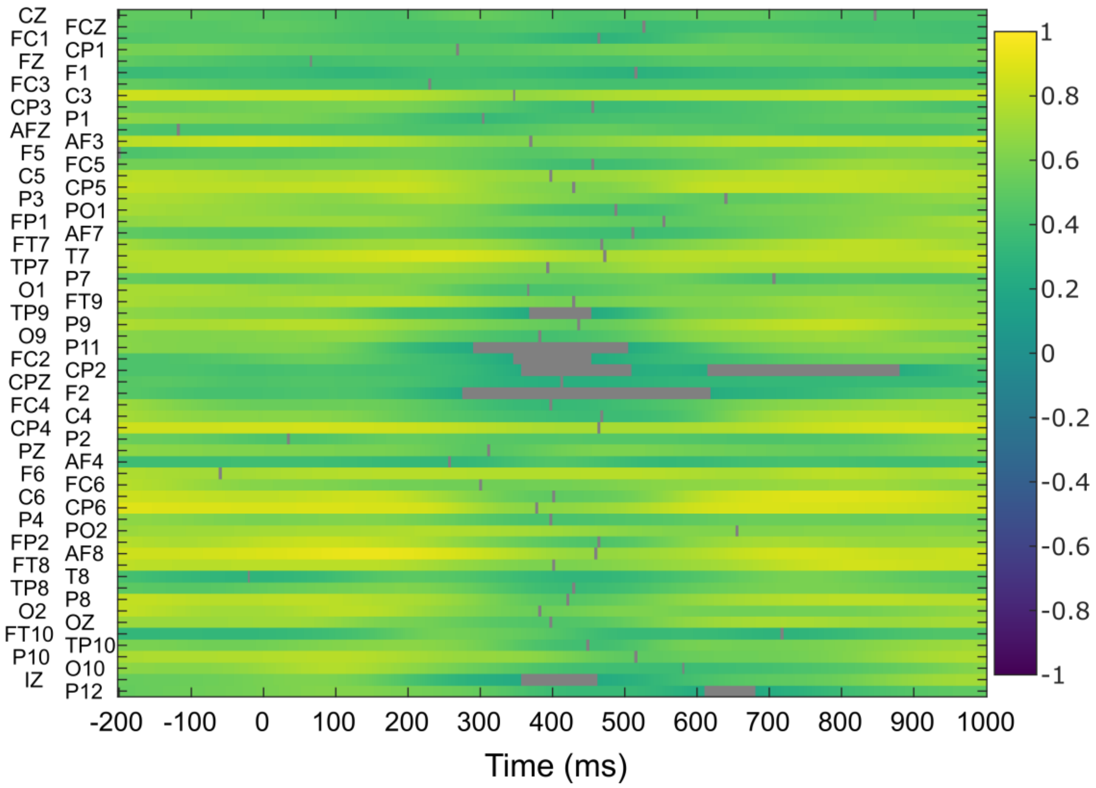

Figure S8

Correlation between inhibition-related theta and alpha power in the C-cluster. The figure depicts the correlation matrix for theta and alpha activity at each time point of the EEG signal in the C-cluster from 200 ms before stimulus onset to 1000 ms after stimulus onset for all 60 channels. Only significant correlations with $p \leq .05$ after a channel-by-channel correction with the false discovery rate method are shown. The correlation coefficient r is indicated by color. The grey areas were not significant after FDR correction.

To obtain a more detailed picture of the specificity of effects regarding the relationship between resting-state and inhibition-related activity, we additionally analyzed the correlation between resting-state theta activity and inhibition-related alpha activity for each time point from 200 ms before to 1000 ms after stimulus onset. Figure S5 and S6 show the corresponding correlation matrix for the S-cluster (S5) and C-cluster (S6). The results show that in both clusters there are significant positive correlations between resting-state theta activity and inhibition-related alpha activity throughout the whole time window. This is not surprising, since inhibition-related theta and alpha activity are strongly inter-correlated (see figure S7 for S-cluster and figure S8 for C-cluster). Around the time point of the theta peak and the typical alpha suppression – i.e. the time point of the very specific correlation pattern between resting-state theta and inhibition-related

EEG and theta activity - this correlation is weakest and even not significant for some channels. In general, there is not such a clear and specific correlation pattern with alpha power as with theta power. Additionally, the strength of the correlation between resting-state theta activity and inhibition-related alpha activity is lower (S-cluster: r_{max}= .58; C-cluster: r_{max} = .59) than between resting-state theta activity and inhibition-related theta activity (S-cluster: r_{max}= .75; C-cluster: r_{max} = .75). These results imply that there is indeed a very specific correlation pattern between resting and inhibition-related theta activity. Yet, the neurophysiological processes underlying inhibitory control seem to be interrelated more complexly across different frequency bands. Future research should approach this aspect in more detail.
